# In Vitro Technology in Plant Conservation: Relevance to Biocultural Diversity

**DOI:** 10.3390/plants11040503

**Published:** 2022-02-12

**Authors:** Verena Kulak, Sheri Longboat, Nicolas D. Brunet, Mukund Shukla, Praveen Saxena

**Affiliations:** 1 School of Environmental Design and Rural Development, University of Guelph, Guelph, ON N1G 2W1, Canada; slongboat@uoguelph.ca (S.L.); nicolas.brunet@uoguelph.ca (N.D.B.); 2 Plant Agriculture Department, Gosling Research Institute for Plant Preservation, University of Guelph, Guelph, ON N1G 2W1, Canada; mshukla@uoguelph.ca

**Keywords:** plant micropropagation, Indigenous, biodiversity, social ecological interactions, science and technology studies

## Abstract

Plant diversity is critical to the functioning of human societies, and evidence shows that plant conservation success is driven by integrative approaches that include social and biological factors. Plants have a unique capacity to reproduce asexually, and propagation practices can yield large numbers of plantlets. These plantlets can be used in several ways to fulfil conservation goals including the repopulation of regions with declining densities of threatened species that hold cultural meaning. However, the potential of in vitro technologies in the conservation of plants that hold cultural meaning is understudied. In this paper we focus upon the roles of in vitro technologies in the conservation of plants relevant to biocultural environments and provide an overview of potential knowledge gaps at the interface of in vitro and plants used traditionally, including those meaningful to Indigenous Peoples. We conclude that in vitro technologies can be powerful tools in biocultural conservation if they are deployed in a manner respectful of the socio-cultural context in which plants play a role, but that further research is needed in this regard. We suggest several epistemological points to facilitate future research.

## 1. Introduction

Plants are pivotal to ecosystem resilience and the functioning of human societies, but they are increasingly threatened. The current rate and magnitude of plant diversity loss is partly attributed to how science and technology are used along with other anthropogenic drivers. Plants have a unique capacity to reproduce asexually, and in vitro propagation technology allows one to produce large numbers of plantlets that can be effectively used to repopulate threatened and endangered species with cultural significance [1] Therefore, in vitro propagation can play a role in the conservation of biocultural diversity [2]. However, the benefits and detriments of deploying in vitro technologies in the conservation of biocultural diversity are understudied. Biocultural diversity allows framing the problem of biodiversity loss under socio-cultural and ecosystem components, blurring the human–nature divide. This view acknowledges that the degradation of life’s diversity indiscriminately affects humans and other organisms, and that conservation interventions impact human and ecosystem dimensions. In this review, we discuss key points of the human dimension at the interface of plant diversity loss, in vitro technologies, and biocultural conservation, focusing on plants meaningful to Indigenous Peoples. We describe potential knowledge gaps and current challenges in the human-plant-technology relations, concluding that in vitro technologies are potentially useful tools in biocultural conservation if they are deployed in a way respectful of the socio-cultural context in which plants play a role. We also suggest that future scholarship should include discussion on how to define Indigenous plants, and that these efforts be conducted in consultation with Indigenous Peoples. Respectful and just collaboration can facilitate a shared research space among plant biologists, social scientists, and Indigenous Peoples to better understand the role of in vitro technology in the successful conservation of biocultural environments.

## 2. The Loss of Plant Diversity: Threats, Drivers, and Magnitude

Plants play a pivotal role in maintaining ecosystem resilience, and their diversity is considered critical to the survival of human societies, and yet, plant diversity is increasingly threatened [2,3,4,5,6,7]. Plant diversity, framed here as a sub-theme of biodiversity, is defined as the variability of plant life on Earth at the genetic, species, and ecosystem levels [8]. Biodiversity loss, commonly understood as the reduction of variability from genes to ecosystems, includes the extirpation and extinction of species and the degradation of habitats [9,10]. The loss of diversity has been connected to the weakening of ecological food webs, agricultural decline, and economic losses [7,9,11]. Thus, biodiversity loss can be regarded as a complex problem with no linear solutions and as a common denominator to biological and societal challenges [6,12,13]. Biodiversity loss is a global phenomenon occurring despite numerous conservation initiatives and innovations initiated since the 1970s, when the importance of biodiversity loss was formally acknowledged [9,14,15]. Although there is no wide consensus on the metrics to assess biodiversity loss, current scientific studies and global policy reports agree that anthropogenic factors such as human population growth, current rates of natural resource exploitation, and pollution are important drivers of the contemporary rate of this loss [5,11,12,13]. The current loss of plant diversity has been triggered by land use changes initiated by deforestation, desertification, intensive monocultures, invasive species, and urban sprawl [14,15]. Furthermore, the interrelations between humans and nature have supported the argument that humanity is driving the sixth mass biological extinction [16]. Anthropogenic actions have increased the rate of biodiversity loss by at least 100 times the background extinction rate i.e., the naturally occurring rate as observed in the geological record [16,17,18]. In terms of plant loss, it is estimated that anthropogenic drivers are causing the loss of one potential medicinal plant species every 2 years. Some species are disappearing before being known to science, and their loss is projected to occur within the next decades [11,19,20]. Given the complexity and wide-ranging negative outcomes of biodiversity loss, involving stakeholders and rightsholders and partnering with local communities offer advantages for identifying and protecting plants of ecological and social significance [2,21,22,23].

Biodiversity loss has also been linked to climate change, which further emphasizes the role of plants and the interactions among diverse traditional knowledge systems, technologies, and social perspectives [24]. Mayhew and colleagues presented the first scientific evidence directly connecting global climatic changes with fluctuations in biological diversity across time, finding higher extinction rates in both terrestrial and marine environments during the warmest planetary phases [25,26]. Although climate change is considered a determinant of biodiversity loss, these two phenomena are now understood as part of a feedback loop in which climate change exacerbates biodiversity loss and vice versa [27,28,29]. While this relationship is observed in nature across the geological scale and pre-dates modern humanity, it is the precipitous nature of the human-triggered climate change and biodiversity loss that underpins the sense of urgency in current scientific and policy matters [20,30]. These two phenomena are inflicting further evolutionary pressures on plants, while at the same time plants are considered fundamental in mitigating the effects of climate change [31,32]. On the one hand, trees are primary producers that act as carbon traps reducing atmospheric CO_2_ and have heat-holding effects, simultaneously supporting heterotrophs. At the human scale, plants provision foods, medicines, and sources of income, which help attain sustainability goals [33,34,35,36]. Thus, projects that focus on plant components for retaining and restoring the integrity of forests and other ecosystems are gaining attention in conservation [28,37]. On the other hand, changes to plant diversity in boreal and high-altitude regions have been found to exacerbate the vulnerability of cold-adapted plant populations, increasing the risk of their extinction [31,38]. Species adapted to cooler environments are susceptible to warmer temperatures and respond to the stressors through in-situ adaptations, including shifting their geographical location from warmer to cooler ranges to avoid extirpation and possible extinction [39,40]. Several plant species that were thought to occupy lower altitudes have been recently mapped at higher altitudes, forcing them to interact with other plant species, soil biota and pollinators [41]. Modelling and mapping studies predict that by the end of the 21st Century, high-mountain wild plants in the Alps, such as the medicinal species *Salix reticulata* L. (snow willow) and *Saxifraga* spp. (saxifrages) may lose up to 40–50% of their present range as they move to cooler environments at higher altitudes [42]. Similar changes have been observed in the Medicine Mountains in the eastern Himalayas, depriving the Tibetan communities of access to an estimated 61% of their unique plant medicines such as *Saussurea laniceps* Hand.-Mazz. (snow lotus) [43]. As plants eventually reach areas with less suitable soil and less room to grow, they compete for limited resources and struggle to re-establish and maintain healthy populations [42,44], which can have negative implications for cultural practices because many plant species are unique sources of medicine, food, and ceremonial artifacts [32,45,46,47]. Therefore, the compounded effects of plant biodiversity loss and climate change weaken the structural and functional interconnections between the biological and cultural components of life [7,48,49]. In this regard, deploying plant-focused technologies, not exclusively as technofixes but as part of integrated schemes, can contribute to safeguarding both biological and cultural diversity [50,51,52].

Conservation is among the activities implemented to deal with biodiversity loss. The term conservation is generally understood as the science and practice of describing, explaining, appreciating, protecting, and perpetuating biological diversity, which is an inherently interdisciplinary and deliberate undertaking [53]. Conservation science has its roots in the western worldview that originated in Europe and assumes that human agency aided by the scientific method and technology can remediate biodiversity loss [54]. Technology refers to the body of knowledge available to a culture or society to design, produce, maintain, and use physical objects (tools, instruments, or artifacts) to extract, collect, produce, or distribute materials, energy, or information, providing a benefit [55,56,57]. The way technology is deployed reflects societal values and priorities, so tool use can carry social meaning [58,59]. The close association between science and technology gave rise to the concept of technoscience, which in the social sciences is useful in analyzing wide-ranging technologies and scientific discoveries such as biotechnology along with the social context in which they operate [60]. Within this scope, the way science and technology are used in conservation is a novel research theme in the field of science and technology studies (STS), a discipline exploring the meanings and interactions between science, technology, societies, and the natural environment [61,62,63]. Several STS concepts are used in the following sections to explain human-technology-plant interactions. But first, it is important to introduce the concept of biocultural diversity to better assess the role of in vitro technology in the conservation of plants that hold biological and cultural value.

### Biocultural Diversity and Plant Conservation

It has been argued that biodiversity also includes cultural diversity, giving rise to the more comprehensive concept of biocultural diversity [23,64,65,66,67]. Cultural diversity refers to the variety of cultures defined as systems of shared symbols, behaviors, beliefs, values, norms, artefacts and institutions that the members of a society use to cope with their world and with one another, and that are transmitted from generation to generation through learning [49,68,69]. The inclusion of the socio-cultural dimension emphasizes that societies, their cultures, and nature are in constant interactions, which are integral to human survival in the biosphere [70,71]. Biodiversity and language loss have also been linked to the loss of unique medicinal plants, uses, and meanings especially among peoples who practice oral and land-based teachings [72,73]. Biocultural diversity conservation aims at sustaining the biophysical and sociocultural integrity of life systems, including the protection of species that reflect long-held relations with nature and help shape cultural identity [52,74]. Furthermore, all technologies including in vitro methods have sociocultural dimensions, and their deployment in biocultural environments requires a deep understanding of their impacts upon human societies and their ways of life [75,76]. In this context, there is a need to build a common epistemological space in which biocultural conservation themes can be approached alongside plant sciences and technologies [77,78].

While all human societies are coupled with the ecologies around them, biocultural diversity loss has disproportionally affected some populations more than others and has been qualified as a crisis among Indigenous communities [74,79]. There is no universally accepted definition of Indigenous Peoples; however, there are some general criteria to identify and distinguish Indigenous Peoples from the dominant society. The United Nations has proposed to refer to Indigenous Peoples as those in independent countries who self-identify at the individual level and are accepted by the community as their member. They are regarded as Indigenous on account of their descent from the populations that inhabited the country or a geographical region to which the country belongs, at the time of conquest or colonization or the establishment of present state boundaries and who, irrespective of their legal status, retain some or all of their own social, economic, cultural and political institutions [80]. Indigenous Peoples have developed their own unique ways of interacting with their traditional environments [81]. Although a generalized Indigenous system does not exist, Indigenous knowledge systems (IKS) are not just knowledge but encapsulate a way of life [82]. IKS are based on the beliefs, assumptions, and understandings of non-western people developed through long-term associations with a specific place offering a holistic (i.e., interconnectedness of the cosmos) view of the world and of themselves [78]. Therefore, IKS are inseparable from Indigenous Peoples, who maintain the relationships with living human and non-human entities, spirits, ancestors, and future descendants [82]. Indigenous languages can also reflect and help perpetuate intimate practical environmental knowledge and intrinsic values that contribute to biodiversity protection and its sustainable use within IKS [81,83,84]. In this view, Indigenous Peoples have advocated the use of a holistic conservation perspective informed by local knowledge while also considering the potential of western technoscience [85,86]. Some Indigenous experts argue that the integration of Indigenous knowledge and western-rooted technoscience can occur under approaches such as the Two-Eyed Seeing, or Etuaptmumk in Mi’kmaw [87]. This refers to a view informed by two lenses used together without exerting dominance to generate solutions that are good for people and the environment [88,89]. Responsible and just bilateral approaches such as Two-Eyed Seeing can be advantageous in plant conservation because they consider that many plants encapsulate sacred and linguistic values as well as being foundational to ecological functioning [90,91].

In plant conservation, biotechnology including in vitro methods offers advantages in propagating and storing plant germplasm especially benefiting species for which seed-based or other conservation methods are ineffective [92]. However, biotechnology in conservation has had varying degrees of success and social acceptance [93]. This is in part because the processes behind how the technology is perceived, implemented, regulated, and funded at cross-cultural scales remain little understood and documented [94]. In this context, the conservation of the American chestnut (*Castanea dentata* Borkh.) presents an interesting example. This tree is the first plant species to undergo genetic engineering for conservation using a wheat gene resistant to chestnut blight [95]. The goal is to reintroduce the species into the wild. American chestnut was a dominant species in eastern North America but is now functionally extinct in the United States due to the accidental introduction of the fungus from Asia in the early 1900s [96]. However, Hodinöhsö:ni’ (Haudenosaunee) communities inhabiting lands where now-extinct chestnut trees used to exist were not initially consulted on the use of genetically engineered organisms (GMOs) for chestnut restoration [97]. Collaboration is encouraged in the UN Declaration on the Rights of Indigenous Peoples, but consultation is often carried out after designing or conducting laboratory experiments [1,96]. However, the modified chestnut trees are projected to be re-introduced in environments shared with Haudenosaunee Peoples [97]. This is an issue of concern for some Haudenosaunee Peoples because in their view, a new kinship bond would need to be built between people and the new trees [93]. This illustrates that technological introductions can engender socio-cultural tensions or alter human–plant relationships that can be counterproductive to the success of plant conservation and the wellbeing of people [73].

## 3. Plant Conservation and Social Dimension of Technoscience

In STS scholarship, it has been argued that technology mediates the interactions between humans and their environments, which can be beneficial or detrimental [48,75]. Mediation in this context refers to facilitating certain interactions over others and to helping shape understandings about the world that would not happen in the absence of technology. This means that tools are not simply passive objects that lack influence over humans and the world, which in the case of this review refers to the human–plant relationships [59]. For example, tools allow humans to transform entire landscapes at rates much higher than by using hands alone. Simultaneously, the transformed environments and the tools themselves change the way humans behave, because tools enhance abilities and widen the possibilities of using plants [98]. However, there are several unknowns in the human-technology-plant relationships due to slower progress in plant conservation scholarship compared to that of animals [5], and due to limited evidence on the social aspects in the technoscience-plant relationship [99]. These two issues are discussed in the next section.

### 3.1. Studies on the Conservation of Plants: Limitations and Opportunities

There are two socially related issues in the conservation of plants. First, scholars have argued that plants take a backseat to animals as subjects of study, and as funding and policy targets. Unsurprisingly, most charismatic species framed in conservation are animals such as the orangutan (*Pongo pygmaeus* L.). This species has directed global attention to Borneo while orchid species, many of them endemic, have not been fully identified in that region [100]. This neglect has been called plant blindness and continues to happen regardless of calls for action by scholars such as Amos [6], Thuiller et al. [31], and Sharrock et al. [101]. Further, Wandersee points out that specific social attitudes and conservation targets need to be developed to protect plant species [102]. Plants are generally perceived as non-sentient beings, so people do not feel empathy for them [103,104]. In conservation practice, empathy towards pain and suffering plays an important role in conveying a public message of protection placing animals in an advantageous position over plants [105]. The view of plants as capable of experiencing pain is hard to reconcile with a western biological view because pain is generally defined as a neural response. Plants do not have neurons, thus pain cannot be observed or measured with the tools used for animals with nervous systems [104]. Second, in conservation science, policy, and law, plants are generally seen either as resources or as components of nature that hold intrinsic value [6]. In either case, plants may or may not warrant protection based on what is prioritized and by whom. Margulies et al. argue that the relative neglect given to plants in conservation along with limited views of plant-human relationships have contributed to lax legal control and the indiscriminate illegal trade of many species [106]. Plants viewed only as tangible resources are often illegally traded for essential oils, perfumes, exotic foods, herbal remedies, and ornamentals, increasing the threat of their extinction. This view can also cause human casualties in what Walters [107] defines as eco-crimes such as targeted assassinations of locals and environmental activists who inhabit coveted lands or oppose resource exploitation [108,109]. When these are Indigenous plants, it can lead to the deprivation and extinction of related experiences among Indigenous Peoples [96]. Therefore, how plants are interpreted can pose limitations or advantages in conservation.

In this regard, there is a third view of plants that needs consideration. Their behavior can be interpreted as resulting from agency, which attributes an active social role to plants. In ethnobotany and folk psychology, agency generally indicates the ability of an entity to act and influence other actors in the social world [110]. In biology, plant behavior such as growth towards sunlight is understood as a biochemical process. In contrast, in several IKS, plants participate in networks of relationships with humans and other components of the land [90]. For example, the Ngobe People, an Indigenous group in Panama, consider plant growth as a means of communication with other entities including people, which indicates plant agency. Growing towards sunlight or shedding seeds are interpreted as feelings such as happiness and are expressed by specific Ngobe words. On the contrary, if the plant is not shedding seeds in season, the plant is said to be in pain. Relating to plants in this manner has allowed the Ngobe to reduce the harvesting of certain plants during drought years [111]. Although western scientific research about plants is widening, framing plants as social participants or as capable of feeling places western plant biologists and policymakers in uncomfortable epistemological situations [6,104]. Nonetheless, plant scientists who intend to deploy in vitro technology in conservation should be aware that the socio-cultural roles of plants in IKS diverge from western technoscientific visions [51,110].

### 3.2. Indigenous Plants

Ethnobotanical data has increasingly been incorporated in conservation scholarship denoting an awareness of the socio-cultural roles of plants. Framing plants as biocultural components demands a working definition of Indigenous plants. In plant sciences and biogeography, native, endemic, or indigenous (i, not capitalized) refer to plants that are present in a region due to natural evolution and dispersion, as opposed to invasive plants that are introduced by anthropogenic means outside their native ranges [111]. This definition does not capture Indigenous plants (I, capitalized), loosely defined as plants that hold traditional meaning to Indigenous Peoples. There are Indigenous plants that are not indigenous to regions of cultural significance but that have been used traditionally for many generations. For example, maize (*Zea mays* L.) was originally domesticated in central Mexico, but its cultivation and cultural meaning disseminated to North and South America, becoming a traditional plant and strong symbol of identity among numerous Indigenous groups across the Americas. For the Haudenosaunee, maize is one of the three traditional crops along with beans and squash, comprising the Three Sisters [112]. However, maize is a domesticated crop that under a biogeographic definition can be interpreted as an invasive species in northern and southern regions outside central Mexico; hence, it could be excluded from ecological conservation efforts. However, from a biocultural perspective, maize is an Indigenous plant of paramount importance to food security and the perpetuation of traditional lifestyles. Due to high hybridization, maize is a very vulnerable crop and introgressions with its wild relatives, the teosintes (*Zea* spp.), have been of interest. Even if maize is not a focus of conservation, wild teosintes are all considered threatened [113]. Protecting the biodiversity of the domesticated and wild forms is, therefore, relevant in biocultural conservation. This case exposes some of the challenges of generating definitions that work at the interface of western technoscience and IKS. Unfortunately, scholarly discussions on how to accurately define “Indigenous plants”, to the best of our knowledge, are rare. In ethnobotanical literature, terms such as traditional, medicinal, or sacred are commonly used to describe socio-cultural roles of plants, but these terms do not appear to have been formally reviewed in conservation literature [109,114,115]. Traditional is a term used informally and repeatedly in this review to denote actions or events that are conventional, customary, and long-established, and which do not refer exclusively to Indigenous contexts. We suggest that a discussion with Indigenous partners about how to define Indigenous plants is warranted to capture plant meaning, and to create a common language in biocultural conservation.

### 3.3. Social Dimension, Technoscience and Plant Relationships: Concerns

The outcomes from the deployment of conservation technology in Indigenous social contexts are understudied [75,116,117], but hold potential benefits. For example, in Mexico, several cacti of *Turbinicarpus* sp., known as peyotls (peyote) by the Huasteco and Huichol peoples, are considered sacred and medicinal due to their psychoactive properties. At the same time, due to these properties and their ornamental appeal, these small cacti are illegally traded with few legal deterrents [115]. *Turbinicarpus* species have a very slow growth rate, so their populations have been rapidly depleted, leading to inclusion in CITES Appendix 1 and the IUCN Red List [116]. Due to their rarity, traditional rituals using peyote and the unique hand-carved vessels used in rites are also disappearing [117]. Beyond their sacred meaning within IKS, peyote conservation can open opportunities in pharmacology. To this end, several recent studies have documented the use of in vitro propagation to produce specimens for non-Indigenous use and to divert the exploitation of wild populations [118,119,120,121]. However, the use of Indigenous plants by non-Indigenous peoples is a contentious issue, so harvesting or manipulating plants must occur with just and respectful consultation [1,76,96,97]. In sum, in vitro propagation technology can sit at the interface of Indigenous and non-indigenous plant use, but its deployment must be guided by socio-cultural narratives.

## 4. The Nexus of In Vitro Technology and Plant Conservation

Dating back about 10,000 years, humans began using living organisms to obtain products and solve problems, for example, animal or plant domestication, giving rise to biotechnology [122]. Biotechnology exploits a facet of a life cycle or a trophic relation between organisms and includes selective breeding of plants [123]. At present, as a technoscience, biotechnology is largely performed in laboratories and other controlled environments using specialized equipment to observe and manipulate organisms. The term in vitro, meaning in glass, is widely used in the life sciences [124]. In vitro technology for plant production focuses on regenerating whole organisms from tissues and cells, producing genetically identical specimens or clones. This technology manipulates plant components, called explants, by isolating them from their natural environment and stimulating them to grow in receptacles assisted by aseptic growth media, controlled light, temperature, and other conditions [109,122,123,124]. Seminal work on plant tissue culture was published in 1902 by Gottlieb Haberlandt, who predicted that artificial embryos (human-made) could be cultivated from vegetative cells in test tubes, culture boxes or Petri dishes [125,126,127,128,129]. Later the ability to grow and multiply isolated shoots laid the foundation for large-scale propagation of plants. Today, this technology has wide applications in agriculture, pharmacology and increasingly in conservation [109,121].

### 4.1. Micropropagation and Cryopreservation Methods

**Figure 1:**
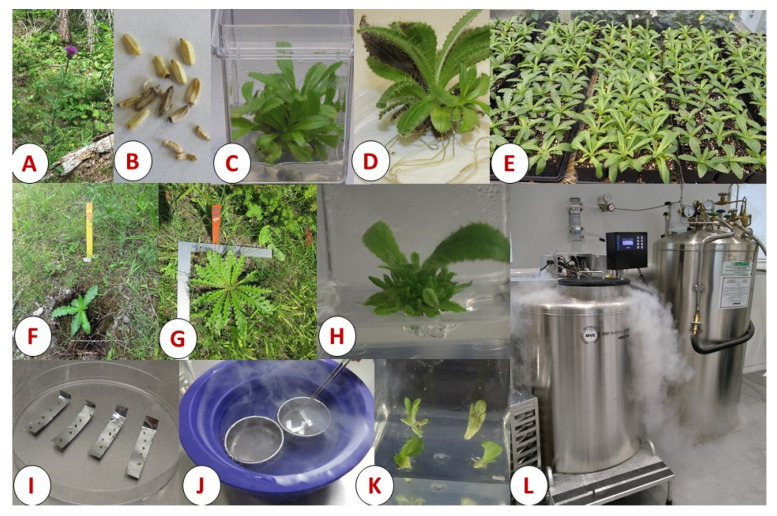
Illustration of various steps in micropropagation of plants and their long-term storage in cryopreservation. Hills’ thistle plants (**A**) growing in their natural habitats (Tobermory, Ontario, Canada) and seeds (**B**) were collected from Parks Canada, Tobermory to initiate in vitro culture from germinated seeds. Multiple shoots (**C**) and rooting (**D**) of Hill’s thistle micro-shoots developed through micropropagation; rooted plants were acclimatized under greenhouse conditions (**E**). Acclimatized plantlets reintroduced in their natural habitat (**F**), and normal plant growth observed after 1 year (**G**). In vitro shoots of Hill’s thistle (**H**) used to excise shoot tips for cryopreservation through droplet vitrification method, with one shoot in each droplet placed on aluminum foil (**I**) immersed directly in liquid nitrogen (**J**). A surviving shoot tip transferred on shoot growth medium (**K**) for further multiplication and plant regeneration as shown in (**C**–**E**). Many different genotypes of Hill’s thistle and other endangered species as well as economically important crop plants can be stored for decades in a cryobank (**L**). Images taken by M. Shukla.

In vitro technology of plant propagation includes a wide range of modifications and practices such as micropropagation, somatic embryogenesis, slow-growth storage, and cryopreservation, which can be used in integrated schemes (Figure 1). Micropropagation refers to multiplying plants from tissues of wild-harvested or seed-grown plants with the correct combination of nutrients and growth regulators. Regeneration from cultured explants occurs via two different developmental pathways: organogenesis or somatic embryogenesis. In organogenesis, multiple shoots emerge from explants that can be rooted to develop whole plants (Figure 1), whereas somatic embryogenesis produces a bipolar structure that resembles an embryo capable of forming an entire plant. Regardless of the mode of regeneration, both processes are advantageous in producing numerous uniform and healthy plants of desired genotypes with traits such as pest resistance, stress tolerance, high medicinal content, etc. In addition, these methods may allow one to recover healthy plants from those infected with parasites and pathogens [130,131]. For conservation, micropropagation has been successfully used in the reintroduction of endangered species into their native ecoregions. A few examples of the use of micropropagated plants in conservation include (a) the medicinal plants *Achillea occulta* L. (yarrow), *Amsonia orientalis* Decne. (European bluestar), *Anthyllis splendens* L., and *Calamintha cretica* Mill. (calamint) in Greece [132]; (b) *Prunus africana,* Hook f. (African cherry), a medicinal tree, and the ornamental *Magnolia sirindhorniae* Noot. & Chalermglin [133,134]; and (c) *Cicer microphyllum* Benth. (Himalayan chickpea), an endemic wild relative of the common chickpea (*Cicer arietinum* L.) [135].

Cryopreservation allows for long-term tissue storage in cryo-tanks containing liquid nitrogen (LN, −196 °C), and the subsequent regeneration of plants from these tissues following rewarming [126,136,137]. Under cryopreserved conditions, explants are stored in a state in which cellular divisions and metabolic activities are minimal, thus preserving the genetic integrity for potentially indefinite time [126,138,139]. The process of freezing plant tissues in liquid nitrogen requires substitution of water content in the tissues with cryoprotectants that are anti-freezing substances capable of inhibiting ice formation and protecting cellular structure. Cryopreservation protocols are often specific to each plant species [136]. Commonly used explants such as meristems, nodes, buds, roots, and seeds can be used for plant species with irregular seed production and for species in which seed collection is limited due to dwindling populations [137,140]. Cryobanking has been used for plant reintroductions in natural habitats in species such as golden paintbrush (*Castilleja levisecta* Greenm.) [141], cherry birch (*Betula lenta* L.) [142], and the critically endangered pearl-like androcalva (*Androcalva perlaria* Wilk.) [143]. Recently, Streambank lupine (*Lupinus rivularis* Lindl.), an endangered plant in Canada, has also been micropropagated and cryopreserved successfully [144]. Table 1 shows several traditional plants in which micropropagation and cryopreservation have been applied for conservation purposes.

### 4.2. Advantages of In Vitro Technology

In vitro technology for plant conservation generally promotes the protection, reintroduction, and restoration of species in cases where seed banks or traditional propagation are not sufficient or adequate. Introducing clones from another population into isolated populations can contribute to genetic rescue in conservation by increasing genetic variability, although this issue needs further study [167]. Using clones made from selected mother plants has the goal of replenishing dwindling or extant populations. Although research is ongoing on the role of clones in conservation, asexual reproduction remains a widespread strategy observed in plant reintroductions [131,168]. There are three main applications of in vitro technology in conservation:

1. In vitro methods facilitate tapping into the abilities of plant tissue to reproduce vegetatively from limited starting material, thus reducing the need to harvest whole plants or numerous plants from the wild, preventing the depletion of vulnerable populations in their natural habitats. Plant multiplication by in vitro technology is achieved by proliferation of the apical or axillary meristems, which consist of rapidly growing cells, are generally genetically consistent, relatively virus-free, and bear greater capacity for multiplication compared to non-meristematic tissues. Alternately, plants can be propagated by regeneration, in which individual plant cells express their inherent capacity, referred to as “totipotency”, to divide and differentiate to form complete plants. Both modes of plant propagation have specific advantages. While multiplication using pre-existing meristems is known to produce genetically identical clones that can be used to enrich a specific population, the plants produced by regeneration may exhibit genetic variations that can be exploited to create genetically diverse plant populations. Thus, in vitro technology allows regeneration of fully functional specimens from small amounts of tissue, saving time and money and reducing the need to harvest numerous specimens. The mass production of rare or useful plants via micropropagation diverts from overexploitation of wild specimens for commercial use [131,169].
2. In vitro grown specimens of threatened or rare plants can also help populate ex situ collections in botanical gardens and other research institutions that can be later re-introduced in natural habitats [92,170,171]. This allows for the short and long-term storage of germplasm, which protects it from current threats, and also facilitates the selection of genotypes for future use [172].
3. These techniques allow for an unlimited amount of explant production to supply material for scientific experiments, allowing one to replicate tests ad-lib under rigorous standards and to engage in large trial-and-error conservation interventions [129,173,174].

**Figure 2:**
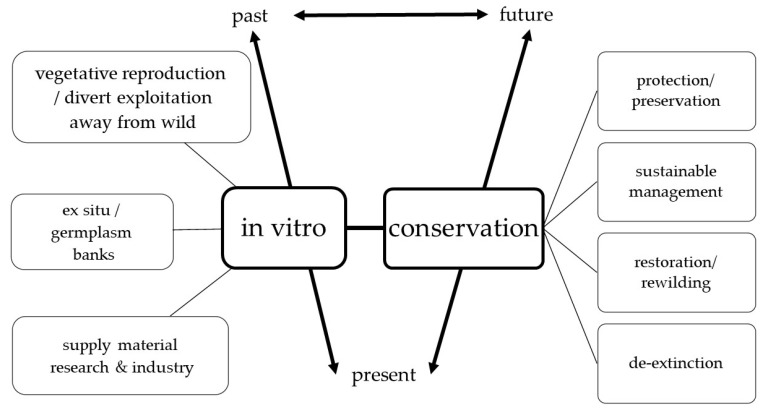
In vitro technologies are applicable in all four conservation outlooks described in this review, as they can assist in protecting, reintroducing, and reconstructing degraded habitats with timelines focusing on the past, present, or future. The interactions between all components have social and biological outcomes. Image by V Kulak.

Conservation in general has never been without disagreement about what to prioritize, and this has produced several research approaches to reverse the loss of biodiversity [53]. Overall, conservation aims at removing existing threats, preventing further negative impacts, reinforcing remaining populations and restoring ecological processes [53,175]. These processes generally translate into four main approaches that reflect which phenomena or conditions are prioritized: (a) the protection and preservation of existing species or habitats from direct human-led harm, (b) the sustainable management of species or habitats that are considered resources for humans, (c) the restoration of threatened species populations or habitats to viable and functional conditions, including reintroduction of extirpated species (re-wilding), and (d) the re-birth or de-extinction of species that have gone extinct along with the re-creation or emulation of disappeared habitats, so species can be brought back to viable self-preserving populations in self-sustaining habitats [53]. These approaches are not exclusive of each other; for example, management and preservation can be implemented simultaneously as part of larger restoration projects. Moreover, preservation is a concept used at several scales, from preservation of intact natural spaces or full landscapes to the preservation of isolated genetic material, tissues, or seeds in vitro or in seed banks [176]. These four main approaches relate to several conceptualizations of interventions across timelines. In other words, they focus on past, present, or future visions of natural environments that can be achieved through human agency. On the one hand, they emphasize cataloging, protecting, or restoring what is present today or in the recent past. This is done by tapping into technologies for ex-situ or in-situ conservation including monitoring and demarcating protected areas. Moreover, this includes maintaining botanical gardens, herbaria, seed banks, and in vitro collections [40,125,139,177]. On the other hand, de-extinction and re-wilding projects rely on cryopreserving specimens to be reintroduced in the future or use genetic manipulation to restore deep-past environments [177,178,179]. Figure 2 depicts in vitro technologies that are applicable in all these strategies and timelines [92,139,140]. For example, preservation of germplasms in controlled environments and cryopreservation intend to capture present diversity as insurance in case natural populations degrade due to harsh anthropogenic conditions.

### 4.3. Limitations of In Vitro Technologies

There are also some limitations and disadvantages of in vitro technologies. For example, it can be difficult to extrapolate adaptation success from results obtained in wet labs back into in vivo and wild environments, particularly because plants in the wild survive along with prevailing abiotic factors and microbiomes including a range of bacteria and fungi and the coping with predators. Thus, conservation projects can require long, multi-season or multi-year timelines [131,180]. Additionally, each species needs to be studied individually to find the optimal conditions necessary to survive and grow in vitro, with a few reports warning that the composition of micropropagation media and supplementation with growth regulators can alter morphological, physiological, and genetic integrity in some plants [125]. Further, in vitro culture is a reductionistic practice because it isolates explants from their original ecological context. Thus, the complexity of plant interactions bound in ecosystems or social networks is minimized [77], as is the case with many scientific studies that isolate components to minimize uncertainty [60]. Plant processes in this context are alterable by one-directional human-to-plant influence, dismissing the possibility that plants hold agency.

### 4.4. Challenges and Opportunities at the Interface of Plant Conservation, In Vitro Technology, and Indigenous Plants

In biocultural conservation, reductionism and control of life processes can challenge holistic visions of the world. For example, in the case of the genetic modification of American chestnut, the Haudenosaunee community is concerned about the kinship ties that need to be developed with the newcomer tree and not merely about the form of the tree [96]. Barnhill-Dilling and Delborne speculated that laboratory manipulation may strip the healing attributed to non-modified (or original, natural) trees, making the transgenic trees suitable only for food or carving [1]. Determining who may access in vitro-raised plants as well as who benefits from them can be a contentious issue, especially when focusing on endangered and endemic plant species meaningful to Indigenous Peoples. The Nagoya Protocol in 2010 was adopted to make explicit the fair and equitable sharing of benefits arising from biotechnology at the global scale [181]. Nonetheless, the mining of plant knowledge from Indigenous Peoples without just and culturally appropriate benefit sharing continues for many endangered medicinal species [36]. Although in vitro technologies require less plant material at the harvesting stage, benefit sharing is in many cases still lacking, especially in countries where the Nagoya Protocol has not been ratified or is simply ignored [182]. For example, green criminology or the illegal trade of endangered species such as orchids is underreported and understudied [183]. Illegal harvest of specimens occurs through in vitro operations to produce seedlings or explants, which are then exempted from CITES control to procure the rare orchid market [184,185]. On a positive side, tissue analysis of suspected plants can be used to trace provenance to enforce conservation protocols [186], and the availability of micropropagation and cryopreservation for many orchid species contributes to their long-term conservation [187]. This illustrates how biotechnologies can sit on both sides of conservation efforts. It means that conservationists operate under ever-shifting biological and socio-cultural contexts where technology can play contradictory roles [188].

One well-documented case of successful use of in vitro technology in collaboration with Indigenous communities is from the Peruvian Andes. Quechua farmers, whose cultural practices reflect an intimate relationship with the highland biodiversity in the Andes, are embracing in vitro techniques to better understand gene flow across domesticated and wild potatoes in the midst of climate change [40]. These farmers, relying on their traditional knowledge, have cultivated thousands of potato varieties (*Solanum* sp.), but pests adapted to warmer climates are now creeping up the mountains and devastating both crop and wild potato diversity. For the Quechua, the spirit of the potato is sacred and acting as stewards of its diversity is ingrained in their identity. Thus, collaboration with in vitro scientists is seen as a positive strategy [162]. In Mexico, several efforts are on-going to optimize tissue culture for several peyote species, so bioactives can be obtained from hairy roots to support pharmacology research while leaving wild specimens intact [118,189]. In Patagonia, several projects have been developed in consultation with the Mapuche to restore *Araucaria* spp. populations via seed banks and vegetative means. However, the potential of in vitro tools remains understudied, and the success rate of plantlet establishment in the wild is still unknown [190]. In the Thar Desert in India, another environment highly susceptible to climate change, a unique collaboration between the Indian government and local communities has been developed to protect endemic species by combining traditional practices with in vitro technologies. Specimens harvested under the supervision of local groups are grown in laboratories to fulfil local needs while diverting the harvest of threatened wild populations [191,192].

## 5. Final Considerations about In Vitro Technology in Biocultural Conservation

Although the social and natural sciences have evolved through different paradigms and methodologies with respect to the approach to conservation, there is growing consensus that including the human social dimensions of conservation is critical to intervention success [193,194]. Conservation projects that ignore the socio-cultural dimensions can unintentionally end up with negative consequences for biodiversity. Responsible and just collaboration between technoscientists and Indigenous Peoples is growing, but there are challenges in reconciling biological and socio-cultural conservation goals. Biotechnology in general is reductionistic and dependent on controlled environments, making it exclusionary or inclusionary depending on the social context in which it is deployed [195]. In vitro technology includes a wide variety of practices that require specialized personnel, instruments and facilities that can be inaccessible to Indigenous communities. Nonetheless, some practices can be transferred and adopted with the limited resources available [196]. Lastly, awareness of the human-technology-plant interactions can guide biocultural conservation strategies and lead to innovation, but the social implications of technology deployment need further study.

## Figures and Tables

**Table 1:** Examples of plants that hold meaning to Indigenous Peoples for which in vitro methods have been applied. We avoided the label “Indigenous” and chose instead “traditional uses” in column two to denote social and cultural roles, because the cited references do not define Indigenous.

Scientific Name	Traditional Uses	In Vitro Method Used	Geographical Location	Reference
*Castanea americana*	food, wood	transgenic * modification *	Northeastern USA	[145]
*Turbinicarpus* sp.	medicinal, ceremonial	tissue culture *^	Mexico	[118]
*Gentiana kurroo*	medicinal	shoot culture *	India	[146]
*Eucalyptus* spp.	medicinal	tissue culture *	Australia, Tasmania	[147]
*Rhinacanthus nasutus*	medicinal, dye	tissue culture *	Southwest Bengal	[148]
*Gethyllis multifolia*	medicinal	hydro culture *	Worcester, South Africa	[149]
*Agathosma betulina*	medicinal, food	micropropagation *	Western Cape, South Africa	[150]
*Wrightia tinctoria*	medicinal	stem cuttings *	India	[151]
*Aristolochia ringens*	medicinal	root, stem cuttings *	Nigeria	[152]
*Manihot esculenta*	medicinal, food	micropropagation *	Global distribution	[153]
*Artemisia tridentata*	medicinal, ceremonial	micropropagation *	Western North America	[154]
*Swertia mussotii*	medicinal, ceremonial	micropropagation *	Qinghai-Tibet Plateau, China	[155]
*Nardostachys jatamansi*	medicinal	micropropagation *	Himalayan region	[156]
*Sequoiadendron giganteum*	ornamental	meristem culture	Sierra Nevada, USA	[157]
*Artocarpus altilis*	medicinal, food	meristem culture *^	Pacific Islands	[158]
*Solanum tuberosum*	food, ceremonial	nodal explant tissue culture *^ shoot tip, micro tuber cryopreservation	Global distribution	[159,160,161,162]
*Hordeum vulgare*	food	embryo cryopreservation *	SW Asia, Himalayas	[163]
*Ceiba pentandra*	medicinal, wood	apical shoot culture	Tropical forests, global distribution	[164]
*Zea mays*	food, ceremonial	embryo, seed cryopreservation	Global distribution	[165,166]

* Indicates in vitro methods have been documented for conservation purposes. ^ Denotes in vitro projects carried out in explicit collaboration with Indigenous Peoples; unmarked reports do not disclose this information.

## Data Availability

Figure 1 was generated from specimens and equipment archived at GRIPP, University of Guelph, Guelph Ontario, Canada N1G 2W1.
